# Supplementary material for: A topological mechanism for robust and efficient global oscillations in biological networks
Source: Nat Commun. 2024 Jul 31;15:6453. doi: 10.1038/s41467-024-50510-x (PMC11291491; doi:10.1038/s41467-024-50510-x)
Supplement: Supplementary file 1 — Supplementary Information [file 41467_2024_50510_MOESM1_ESM.pdf]

Supplementary Information for "A topological mechanism for robust and efficient global oscillations in biological networks"

Chongbin Zheng[1,2] and Evelyn Tang[1,2]

[1] *Department of Physics and Astronomy, Rice University, Houston, Texas 77005, USA*
[2] *Center for Theoretical Biological Physics, Rice University, Houston, Texas 77005, USA*

I. ROBUSTNESS OF OSCILLATIONS IN OUR MODEL

In the topological regime ($\rho \gg 0$), our model supports global currents that propagate along the edge of the state space. The edge currents turn out to be robust against changes in the environment that render certain states inaccessible. For example, a limited number of KaiA molecules in solution could prevent the system from accessing the highly phosphorylated states, while a limited number of KaiB could block off hypophosphoryalated states where x and y are small. Despite missing certain states in the lattice, the probability currents in our model can continue to propagate along the new edge of the state space, as illustrated by Fig. 1. The robustness in edge currents could explain how biological systems maintain stable dynamics in the face of changing external conditions.

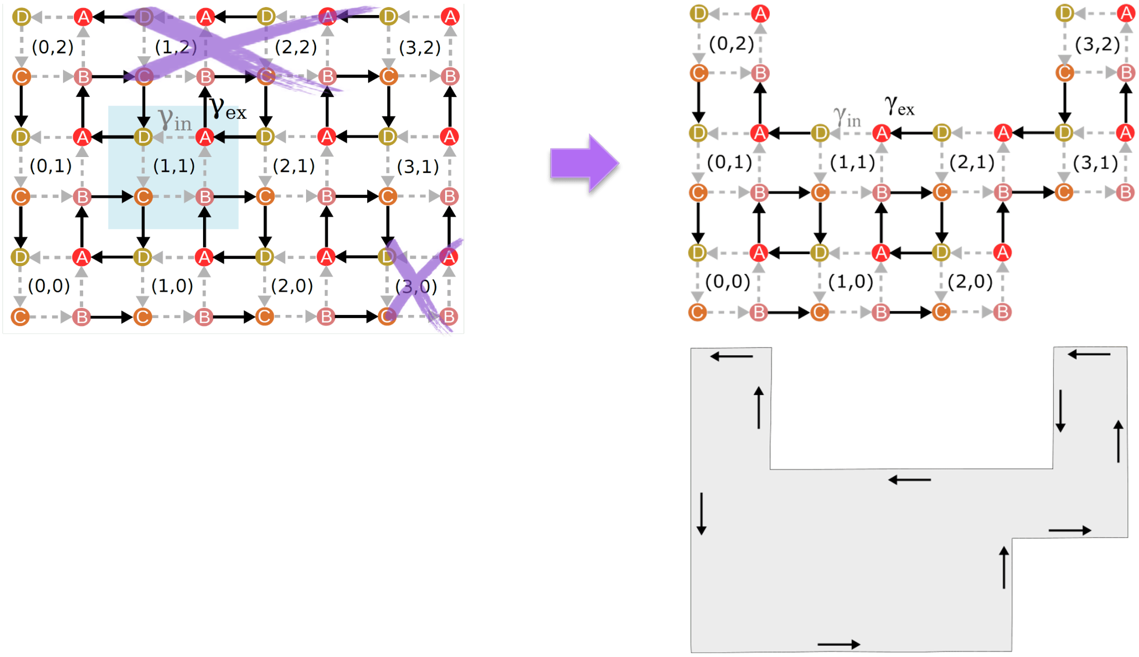

FIG. 1. **Topological protection ensures robustness of the edge state to obstacles or missing components.** In the presence of missing components or obstacles (purple crosses), the edge state (when $\gamma_{ex} \gg \gamma_{in}$) will simply go around them to maintain the largest available phase space. This robustness of the edge state can shed light on how biological systems can flexibly pivot in the presence of changing conditions or external stimuli. For illustration purposes, we use a smaller system with $N_x = 4, N_y = 3$ and only show the forward transitions γ_{ex} and γ_{in}.

The global oscillations in our model are also robust to perturbations in transition rates. The changes in rates can come from changing external conditions like concentrations of KaiA and KaiB or setting more biologically realistic transition rates for each reaction. They can also come from incorporation of additional KaiC reactions hitherto unconsidered. For instance, ATP association with the nucleotide binding site plays the same role as KaiA interaction in promoting T phosphorylation [9]. This reaction can be combined into the S $\rightarrow$ E internal transition, which results in an effective rate for the combined reaction that is larger than the rates for either reactions. To study the effects of such modifications, we make the rates non-uniform in different directions by multiplying each of $\gamma_{ex}, \gamma'_{ex}, \gamma_{in}, \gamma'_{in}$ in each direction (N,S,E,W) by a different random scaling factor f, taken from a normal distribution with mean 1 and standard deviation ϵ. We look at the real and imaginary spectral gaps and coherence of the resulting model, averaged

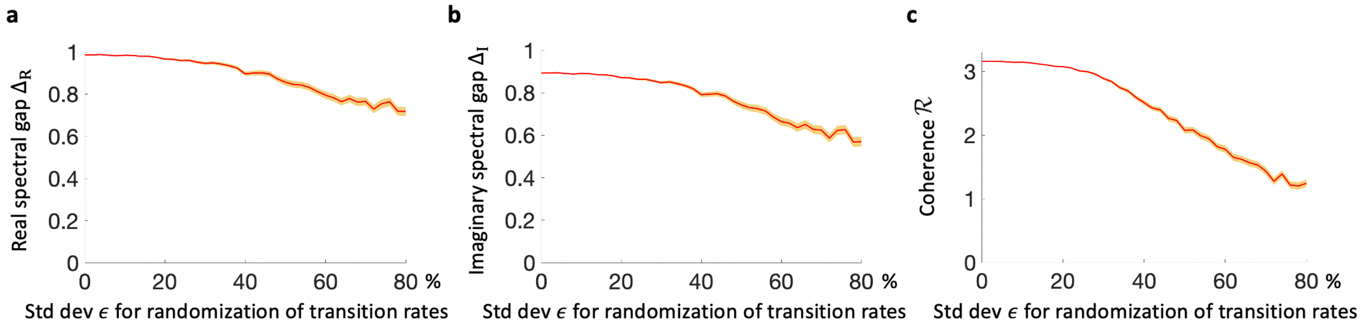

FIG. 2. **Spectral gaps and coherence for randomized transition rates.** **a**, Each transition rate $(\gamma_{\text{ex}}, \gamma'_{\text{ex}}, \gamma_{\text{in}}, \gamma'_{\text{in}})$ in the model is multiplied by a different random scaling factor f in each direction (N, S, E, W). f is taken from a normal distribution with mean 1 and standard deviation ϵ. In the case when a negative value is sampled, we take $f = 0$. Here we plot the real spectral gap Δ_{R}, averaged over different random realizations of transition rates, as a function of the standard deviation ϵ for randomization of the transition rates. The randomized rates repeat along the x and y axis. For example, any eastward γ_{ex} transition has the same rate after randomization regardless of the (x, y) coordinates. **b**, Average imaginary spectral gap Δ_{I} as a function of ϵ. **c**, Average coherence $\mathcal{R}$ as a function of ϵ. For all panels, we average over 500 random configurations for each ϵ. The same configurations are used to calculate all three quantities. The yellow shaded area represent one standard error. The initial parameters for the model before multiplying by f are $\mu = 3, \rho = 5$.

over different random configurations of transition rates. As shown in Fig. 2, the spectral gaps do not close for ϵ up to 80%, so the network is still in the topological regime. In general, the steady state is still localized on the edge of the state space, even though it can have a higher probability in a particular edge, e.g., the left edge, because of the non-uniform transition rates. The coherence of the oscillation is attenuated but some degree of oscillation persists. The robustness of the oscillations obtain from its topological nature, and we expect it to remain robust, up to some extent, for other types of perturbations such as additions of long-range interactions that connect nonadjacent states in the state space.

II. THE 2D ZAK PHASE

Our model topology is characterized by the topological invariant known as the 2D Zak phase [7, 11]. It is defined as an integral over the 2D Brillouin zone:

$$\mathbf{\Phi}_i^{\text{c}} = \frac{1}{2\pi} \int_{\text{BZ}} \mathbf{A}_i(k_x, k_y) dk_x dk_y, \tag{1}$$

where $\mathbf{A}_i(k_x, k_y)$ is the Berry connection for the i-th band, given by $\mathbf{A}_i(k_x, k_y) = i\langle \phi_i | \partial_{\mathbf{k}} | \psi_i \rangle$ for left and right eigenvectors $\langle \phi_i |$ and $|\psi_i\rangle$, respectively. For our stochastic system, we focus on the Zak phase $\mathbf{\Phi}_{\text{h}}^{\text{c}}$ for the highest band h in real space (upper green band in Fig. 4b in the main text), which is the band closest to the steady state eigenvalue of 0. It turns out that when our system is in the trivial regime ($\rho < 0$), we have $\mathbf{\Phi}_{\text{h}}^{\text{c}} = (0, 0)$. When our system is in the topological regime ($\rho > 0$), we have $\mathbf{\Phi}_{\text{h}}^{\text{c}} = (\pi, \pi)$. The emergence of the dynamical regime with edge currents and edge-localized steady state coincides with a nontrivial 2D Zak phase, indicating its topological origin.

III. COHERENCE

In this section we motivate the definition of coherence in Eq. (2) in the main text and discuss why it can serve as a measure for the robustness of oscillations. We also discuss how coherence changes with the parameters μ and ρ.

For a master equation with non-degenerate eigenvalues

$$\frac{d\mathbf{p}}{dt} = \mathcal{W}\mathbf{p}, \tag{2}$$

the general solution is given by

$$p_i(t) = \sum_\nu c_\nu u_i^\nu e^{-\lambda_\nu t}, \tag{3}$$

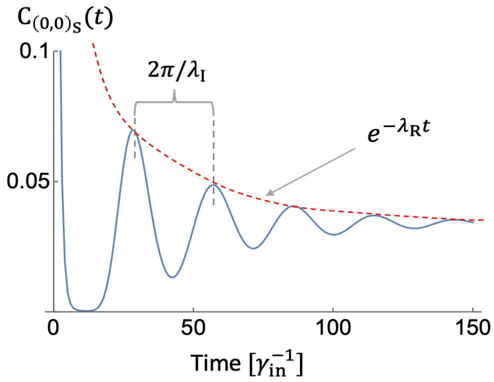

FIG. 3. **Correlation function $C_{(0,0)_S}(t)$ for the topological model goes through damped oscillations.** The oscillation has a decay time λ_R^{-1} and period $2\pi/\lambda_I$. Coherence is defined as the ratio between these two timescales, characterizing the number of coherent oscillations that can be maintained before approaching steady-state. Eventually, $C_{(0,0)_S}(t)$ approaches the steady-state probability p_i^s. Time is plotted in units of γ_{in}^{-1}. The parameters used are $\mu = 7, \rho = 5$.

where λ_ν are the eigenvalues, $(u_1^\nu, u_2^\nu, ..., u_n^\nu)^T$ are eigenvectors corresponding to λ_ν, and c_ν are constant coefficients that depend on initial conditions [10]. The eigenvalues λ_ν of the transition matrix $\mathcal{W}$ characterizes the timescales of the probability evolution for the corresponding eigenmodes. If the network represented by $\mathcal{W}$ is irreducible and ergodic, then there exists a unique steady-state distribution $\mathbf{p}^s$ such that $\mathcal{W}\mathbf{p}^s = 0$, i.e., $\mathbf{p}^s$ is an eigenvector of $\mathcal{W}$ with a zero eigenvalue [10]. The dynamics of $\mathbf{p}(t)$, as we will see soon, is in general dominated by the first non-zero eigenvalue of $\mathcal{W}$, which is the eigenvalue with the smallest modulus in the real part [1]. Such eigenvalues generally come in conjugate pairs, denoted by $-\lambda_R \pm i\lambda_I$ for the real and imaginary parts λ_R, λ_I, respectively. In particular, we take $\lambda_R, \lambda_I \geq 0$ and $\lambda_1 = -\lambda_R + i\lambda_I$.

The dynamics of the system can be studied through correlation functions $C_i(t)$. Following [1], we define $C_i(t)$ to be the probability to find the system at state i at time t given that the system starts at state i at time $t = 0$, i.e., $C_i(t) = p_i(t)$ given the initial condition $p_i(0) = 1$ and $p_j(0) = 0$ for all $j \neq i$. Starting from a state on the edge, say, the lower left corner $(0,0)_S$, the correlation function $C_{(0,0)_S}(t)$ goes through damped oscillations (see Fig. 3). After some transient behavior in the first oscillation cycle, the dynamics is dominated by λ_1, where the oscillation period is given by $T = 2\pi/\lambda_I$ and the exponentially decaying envelope has a decay time λ_R^{-1} [1]. Therefore, we follow [1] and define coherence as

$$\mathcal{R} \equiv \frac{\lambda_I}{\lambda_R}, \tag{4}$$

which, when divided by 2π, is the number of coherent oscillations that can be sustained before the system settles into the steady-state. The larger $\mathcal{R}$ is, the more oscillations the system can maintain before stochastic fluctuations destroy its coherence.

In Fig. 4, we show a phase diagram for $\mathcal{R}$ that include negative values of ρ (the trivial regime). $\mathcal{R}$ turns out to be monotonic in both μ and ρ. $\mathcal{R}$ increases monotonically in μ because a higher μ means a stronger external driving, making the forward reactions dominate more over their reverse reactions. This is more likely to give rise to trajectories with a particular chirality in our model. These trajectories correspond to the robust oscillations observed, and are less likely to backtrack or perform undirected diffusive motion when the external driving μ is large. For ρ, we can see that $\mathcal{R}$ is always close to zero for $\rho < 0$. Large values of coherence is obtained only with positive ρ, even if the thermodynamic force μ is large. This is expected because $\rho \sim 0$ is the transition that separates the topological regime from the trivial regime. In the trivial regime, the system does not support an edge state, and the dynamics resembles random diffusion in the bulk rather than a directed motion along the boundary. Since there are no global oscillations in this regime, the coherence remains low. When ρ is positive and increasing, the system moves deeper into the topological regime, where the edge localization effects are more pronounced, leading to more robust oscillations.

IV. SINGLE-MOLECULE MODELS FOR KAIC

In this section, we include more details on the MWC model and the bilayer lattice model mentioned in the main text. Both models can be represented as directed networks and their dynamics described by corresponding master equations.

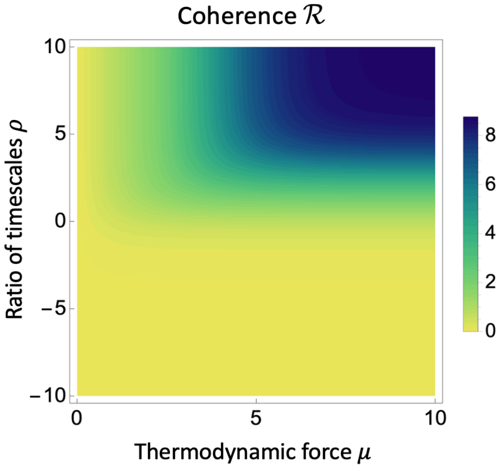

FIG. 4. **Phase diagram for coherence.** The range of ρ is extended from that of Fig. 3a to include negative values. $\mathcal{R}$ is monotonic in both μ and ρ, and remain close to 0 in the trivial regime $\rho < 0$.

The MWC paradigm [8] was first applied to the KaiABC system by Zon et al. [13], which assumed all-or-none conformational change for the entire KaiC hexamer. By assumption, the KaiC hexamer can only be in two conformational states, the active states C_i and the inactive states $\widetilde{\mathrm{C}}_i$, where the subscript $0 \leq i \leq 6$ represents the phosphorylation level of KaiC. There are 14 states in the state space in total. The network structure is shown in Fig. 5a.

In this paper we follow one of the simplest versions of MWC-type models described in [1] and use the same parametrization of the transition rates. When KaiC is in the active state, it is more likely to be phosphorylated. On the other hand, when KaiC is in the inactive state, it is more likely to be dephosphorylated. These dominant reactions are represented by the red vertical arrows in Fig. 5a with transition rates $\gamma e^{\eta/2}$. The reverse transitions (smaller black vertical arrows) have rates $\gamma e^{E/6}$. γ, η, and E are model parameters that can be varied. For the horizontal transitions in Fig. 5a, the rates from C_i to $\widetilde{\mathrm{C}}_i$ are denoted as $k_{i\tilde{i}}$ and the rates from $\widetilde{\mathrm{C}}_i$ to C_i are denoted as $k_{\tilde{i}i}$, where

$$k_{i\tilde{i}} = \begin{cases} 1, & i = 0,1,2,3 \\ e^{E(i-3)/3}, & i = 4,5,6 \end{cases} \tag{5}$$

and

$$k_{\tilde{i}i} = \begin{cases} e^{E(3-i)/3}, & i = 0,1,2,3 \\ 1, & i = 4,5,6 \end{cases}. \tag{6}$$

The value of γ sets the relative timescales between the phosphorylation/dephosphorylation reactions and conformational changes.

In Fig. 5b and 5c we show the network structure for the bilayer lattice model, adapted from Li et al. [6]. The state space consists of two layers of 7×7 lattices that are connected to each other. Fig. 5b shows the network structure of bottom layer from Fig. 5c. Similar to our topological model, the x and y coordinates (labeled by superscripts and subscripts C_y^x in Fig. 5b) represent T and S phosphorylation levels, respectively. The top layer has an identical structure to the bottom layer, with states labeled by $\widetilde{\mathrm{C}}_y^x$. C_y^x in the bottom layer corresponds to unbound KaiC while $\widetilde{\mathrm{C}}_y^x$ in the top layer represents KaiB-bound KaiC. The system can make transitions $\mathrm{C}_y^x \rightleftharpoons \widetilde{\mathrm{C}}_y^x$ between layers while keeping x and y coordinates fixed, which corresponds to KaiB binding and unbinding. To aid comparison, we simplify the model by parametrizing the transition rates with μ and ρ, the same parameters for our topological model. Within both layers, the orientations for faster reactions on each edge are chosen such that they form an overall counterclockwise cycle, to capture the order of the phosphorylation cycle (see Fig. 5b). These phosphorylation/dephosphorylation reactions (solid black arrows) are assumed to have uniform rates γ_{ex} while the slower reverse reactions have rates γ'_{ex}. On the other hand, the KaiB-binding transitions between layers have rates γ_{in} and γ'_{in}. In the upper right half of the lattice where the states are labeled green in Fig. 5b, the dominant reaction between layers is the upward KaiB binding transitions (green dashed arrows in Fig. 5c) with rates γ_{in}, while the downward KaiB unbinding transitions (gray dashed arrows) have slower rates γ'_{in}. This is consistent with the fact

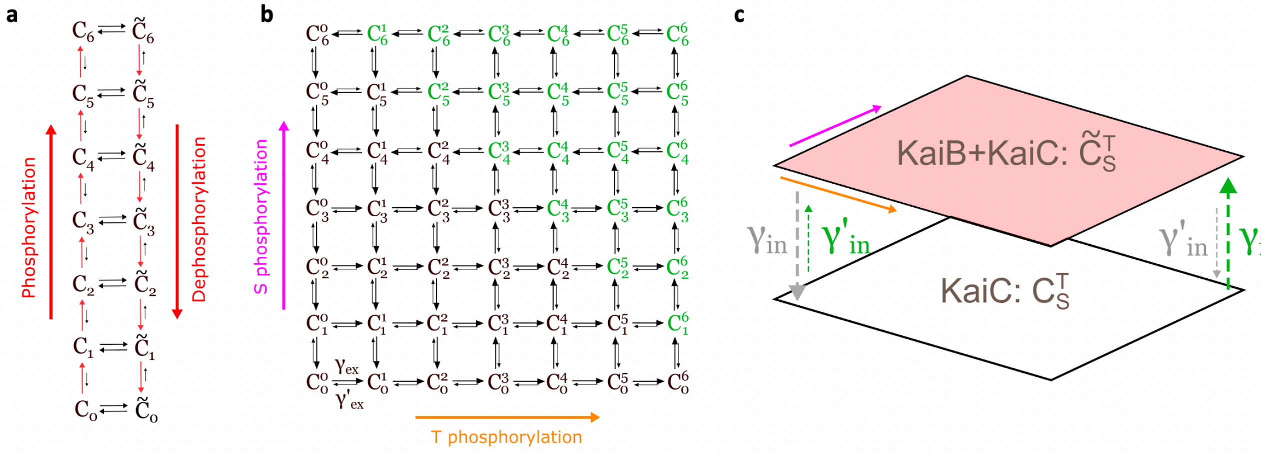

FIG. 5. **Single-molecule models for KaiC.** **a**, The MWC model. The KaiC hexamer can be in one of two conformational states, the active state C_i or the inactive state $\widetilde{C}_i$. KaiC tends to phosphorylate in the active state and dephosphorylate in the inactive state, highlighted by the vertical red arrows. The slower reverse reactions are represented by smaller black vertical arrows. Horizontal transitions correspond to conformational changes of the KaiC hexamer between active and inactive states. **b**, The bottom layer of the bilayer lattice model. Subscripts x and superscripts y represent T and S phosphorylation level, respectively, just as for the topological model. Solid black arrows represent phosphorylation/dephosphorylation transitions with rates γ_{ex} and slower reverse rates γ'_{ex} **c**, A zoomed-out view of the bilayer lattice model. Individual states in each layer are not shown. The green dashed arrows from the bottom to the top layer represent KaiB binding, while the gray dashed arrows from the top to the bottom layer represent KaiB unbinding. For the states colored green in **b**, transition rates are γ_{in} for KaiB binding and γ'_{in} for KaiB unbinding. For the states colored black, transition rates are γ'_{in} for KaiB binding and γ_{in} for KaiB unbinding. Larger dashed arrows (for any color) correspond to faster rates γ_{in} while smaller dashed arrows correspond to slower rates γ'_{in}. During KaiB binding and unbinding, the phosphorylation levels remain the same.

that S phosphorylation promotes KaiB binding [3]. In the rest of the lattice where the states are labeled black in Fig. 5b, the dominant reaction between layers is the downward KaiB unbinding transition with rates γ_{in}, while the upward KaiB binding transitions take the slower rate γ'_{in}. This parametrization scheme gives rise to cycles where a KaiC molecule phosphorylates in the bottom layer, binds to KaiB, gets dephosphorylated in the top layer, and unbinds with KaiB to restart the cycle.

V. COST AND PRECISION FOR DIFFERENT KAIC MODELS

In this section we discuss how the free energy cost (quantified by ΔS) and the precision (quantified by $\mathcal{R}$) of various KaiC models change with the external driving μ. For the MWC model, the entropy production rate σ of the network can be obtained by a cycle decomposition method [10, 1]:

$$\sigma = \eta\gamma \sum_{i=0}^{5} (e^{\eta/2} p^{\text{s}}_{\text{C}_i} - e^{E/6} p^{\text{s}}_{\text{C}_{i+1}}), \tag{7}$$

where $p^{\text{s}}_{\text{C}_i}$ is the steady-state probability at the state C_i. This expression is used to calculate the entropy production per period $\Delta S = \sigma\mathcal{T}$ for the MWC model. As shown in the left column of Fig. 6, ΔS monotonically increases with $\mathcal{R}$, as expected. Coherence, however, is not monotonic in μ. When μ is sufficiently large, the MWC model ends up maintaining less coherent oscillations with increased driving and energetic cost. Note that the $\mathcal{R}$ curve is different from the red dashed line shown in Fig. 3e in the main text. Here we plot $\mathcal{R}$ with (η, E) held fixed, while the dashed line in Fig. 3e shows the maximum coherence for each μ for any parameter combinations (also see Methods). In contrast to the MWC model, coherence for the topological model increases monotonically in μ for a fixed ρ. ΔS, however, is non-monotonic, supporting a regime with increasing coherence and simultaneously decreasing cost (right column of Fig. 6). This unusual regime has its origin from the topological protection of the probability currents on the edge, which effectively reduces the state space into a one-dimensional cycle along the edge. This edge localization leads to a lower free energy cost but more coherent oscillations.

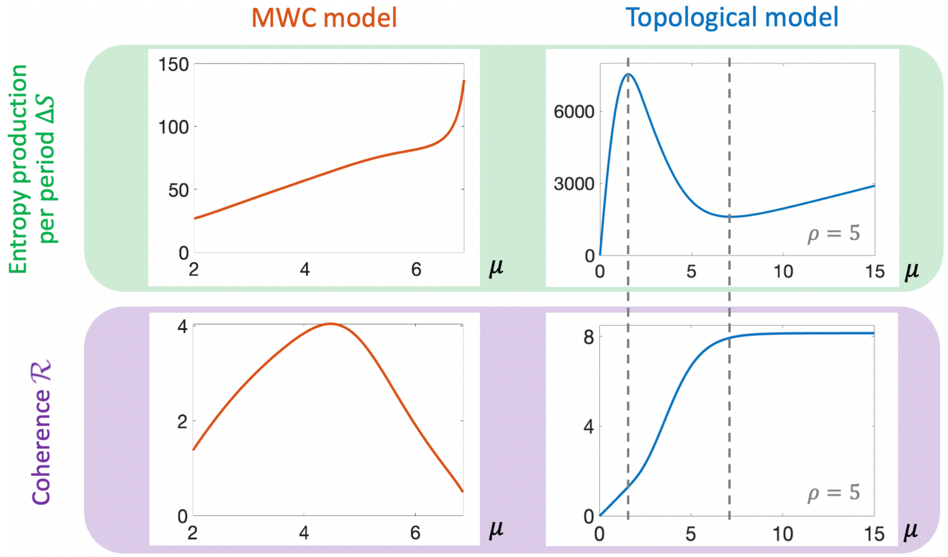

FIG. 6. **Entropy production per period and coherence for the MWC and the topological model.** The MWC model requires more entropy production to maintain a higher coherence, and its coherence decreases as still more entropy is produced under a stronger driving μ. On the other hand, the topological model supports a regime with increasing coherence but decreasing entropy production around $1.5 < \mu < 7$. For the MWC model, the parameters used are $\gamma = e^5$, $E = 10$. We plot μ in a smaller range for this model because outside the plotted range, we have $\lambda_{\mathrm{I}} = 0$, which makes the oscillation period $\mathcal{T}$ and hence entropy production per period ΔS ill-defined. Entropy production per period is given in units of $k_B \gamma_{\mathrm{tot}}$.

VI. SPECTRAL GAP AS A PREDICTOR OF COHERENCE

In this section we study the effects of μ and ρ on the spectral gaps of the transition matrix $\mathcal{W}$ with both periodic boundary conditions (PBC) and open boundary conditions (OBC), by tracking how the eigenvalues change with the two parameters. We also discuss why the spectral gap can serve as a predictor of coherence.

Spectral gaps can be defined for both PBC and OBC. Taking PBC, we can write the transition matrix in reciprocal space as

$$\mathcal{W}_{\mathbf{k}} = \begin{pmatrix} -\gamma_{\mathrm{tot}} & \gamma_{\mathrm{in}} + \gamma'_{\mathrm{ex}} e^{-ik_y} & 0 & \gamma'_{\mathrm{in}} + \gamma_{\mathrm{ex}} e^{-ik_x} \\ \gamma'_{\mathrm{in}} + \gamma_{\mathrm{ex}} e^{ik_y} & -\gamma_{\mathrm{tot}} & \gamma_{\mathrm{in}} + \gamma'_{\mathrm{ex}} e^{-ik_x} & 0 \\ 0 & \gamma'_{\mathrm{in}} + \gamma_{\mathrm{ex}} e^{ik_x} & -\gamma_{\mathrm{tot}} & \gamma_{\mathrm{in}} + \gamma'_{\mathrm{ex}} e^{ik_y} \\ \gamma_{\mathrm{in}} + \gamma'_{\mathrm{ex}} e^{ik_x} & 0 & \gamma'_{\mathrm{in}} + \gamma_{\mathrm{ex}} e^{-ik_y} & -\gamma_{\mathrm{tot}} \end{pmatrix}. \tag{8}$$

We obtain the spectral gaps for PBC from the continuous spectrum defined by $\mathcal{W}_{\mathbf{k}}$, as illustrated in Fig. 4a and 4b in the main text. Similarly, we can obtain spectral gaps for OBC by plotting the spectrum of $\mathcal{W}$ in the complex plane, as in Fig. 7a. For each eigenstate ψ, we also calculate $\sum_{i \in \mathrm{edge}} |\psi_i|^2$, the sum over the squared magnitudes $|\psi_i|^2$ for all entries i that lie on the edge of the system. This quantity characterizes how localized the corresponding eigenstate is on the edge. We normalize each eigenstate so that the value of $\sum_{i \in \mathrm{edge}} |\psi_i|^2$ can vary continuously between 0 and 1. A larger sum, or a redder color, indicates more edge localization. As we can see from Fig. 7a, the OBC spectrum displays two circle-like shapes with eigenstates highly localized on the edge and four clusters of eigenvalues, lying on the real or imaginary axis, with eigenstates more localized in the bulk. These four clusters are identified as the "bands" in the OBC case, in analogy with the four bands in the PBC spectrum as shown in Fig. 4a and 4b in the main text. As shown in Fig. 7a, we define the OBC real spectral gap $\Delta_{\mathrm{R}}^{\mathrm{o}}$ as the shortest distance from the leftmost band to the imaginary axis and the OBC imaginary spectral gap $\Delta_{\mathrm{I}}^{\mathrm{o}}$ as the shortest distance from the topmost band to the real axis.

In Fig. 7b we compare the imaginary spectral gap for PBC and OBC for the same parameters μ and ρ. The two gaps are almost identical at large ρ when the gap is large. When ρ is closer to 0, overlaps between the bands begin to develop for both PBC and OBC, which renders the spectral gap ill-defined. In the regime of relatively large ρ, spectral gaps for the OBC and PBC can be used interchangeably. The same conclusion can be drawn by looking at the two gaps with changing μ and fixed ρ. Therefore, to understand the relationship between coherence and the spectral gap, we can look at the OBC spectrum instead.

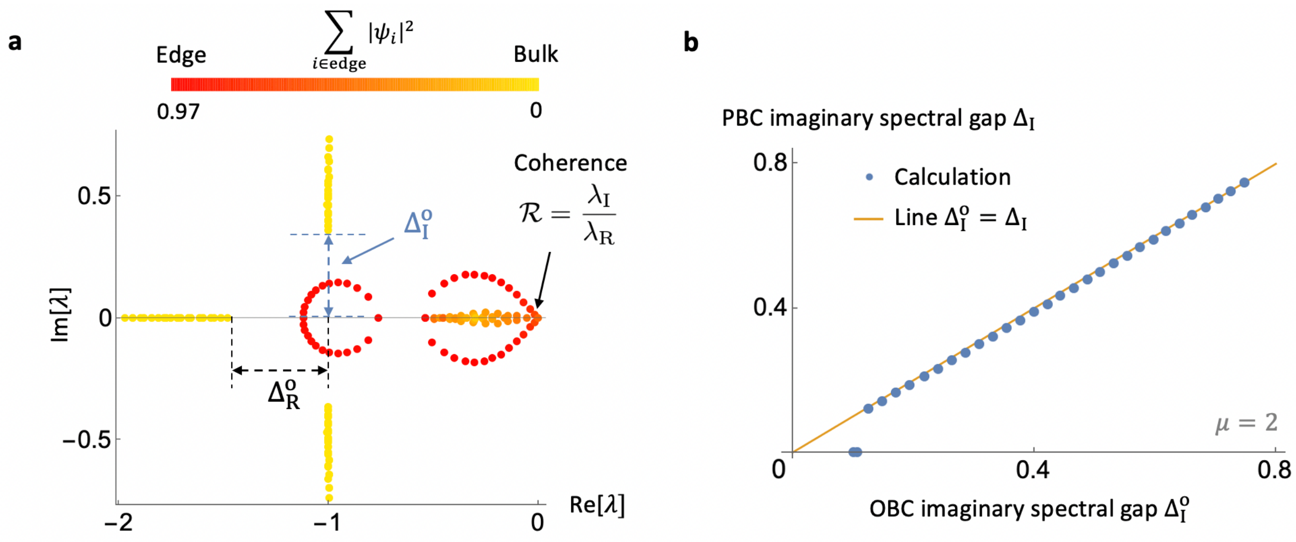

FIG. 7. **Spectral properties of $\mathcal{W}$ in open boundary conditions (OBC).** **a**, Spectrum of $\mathcal{W}$ in OBC. The real spectral gap is defined as the shortest distance between the left band to the imaginary axis, and the imaginary spectral gap is defined as the shortest distance between the top band to the real axis. The color for each dot corresponds to the value of $\sum_{i\in\text{edge}} |\psi_i|^2$, the sum of the magnitude squared of all entries in the steady-state eigenvector that lie on the edge. This quantity measures the extent to which the corresponding eigenvector is localized on the boundary of the system, and lies on a continuous spectrum between 0 and 1. A redder color means more localization. The solid black arrow points at the eigenvalue $\lambda_1 = -\lambda_R + i\lambda_I$, which determines the coherence $\mathcal{R}$. The parameters used are $\mu = 2, \rho = 1$. **b**, The imaginary spectral gaps defined in PBC and OBC are nearly identical when both gaps are large and the bands are well separated. ρ is varied from 0.2 to 4.6 in the plot while μ is fixed at $\mu = 2$. Calculated values for the two gaps (Δ_I in PBC and Δ_I^o in OBC blue dots) almost all lie on the orange line on which the two gaps are equal. Eigenvalues and spectral gaps are given in units of γ_{tot}.

Studying how λ_1 and the entire OBC spectrum change with μ and ρ gives a better picture of why coherence and the imaginary spectral gap track each other. Varying μ and ρ leads to global changes in the spectrum of $\mathcal{W}$ in the complex plane, such as narrowing of the bandwidth or movement of all eigenvalues in a band in the same direction. Meanwhile λ_1, the particular eigenvalue that determines coherence (see Fig. 7a), moves along with the bands in roughly the same way. When ρ is fixed, increasing μ has the effect of increasing the range or dispersion of the spectrum in the imaginary part. When $\mu = 0$, all reactions have symmetric rates with respect to their reverse reactions, and the system is in detailed balance. In this case, the spectrum is entirely real because $\mathcal{W}$ is symmetric. Increasing μ therefore introduces nonzero imaginary parts to the eigenvalues, leading to oscillatory modes with faster timescales as the driving increases. For the top band, for instance, the imaginary parts of all eigenvalues in the band move upwards in the complex plane by roughly the same distance, keeping the bandwidth constant but increasing the distance between the bottom point in the band to the real axis (which is the definition of Δ_I^o). Meanwhile, the real parts of the eigenvalues in the four bands remain virtually unchanged with μ. As shown in Fig. 8a, λ_1 moves in a similar way with the global spectrum. Increasing μ leads to an increase in λ_I but very little change in λ_R. Since $\mathcal{R}$ is proportional to λ_I while λ_R is roughly constant, coherence follows the same functional relationship as λ_I when μ increases (Fig. 8b). Therefore, since Δ_I closely tracks λ_I (see Fig. 4c in the main text), it also closely tracks $\mathcal{R}$ monotonically.

On the other hand, ρ serves as an overall compression factor, decreasing the bandwidth for all four bands. The points farthest away from the real axis for the imaginary bands also increase slightly with ρ. The absolute values of these points equal the sum of the bandwidth for the imaginary band and the imaginary spectral gap. Therefore, a decrease in the bandwidth and an increase in the sum of the bandwidth and the spectral gap imply a monotonic increase in the spectral gap with increasing ρ. λ_1 in this case, however, does not always follow the general movement of the global spectrum. As shown in Fig. 8c, λ_R still decreases monotonically as expected, as the rightmost band is compressed toward the origin. λ_I, on the other hand, develops non-monotonic behavior. Nevertheless, when λ_I begins to decrease, it approaches zero more slowly than λ_R and gives rise to a monotonically increasing $\mathcal{R}$ that tracks Δ_I^o (and therefore Δ_I) in ρ (Fig. 8d).

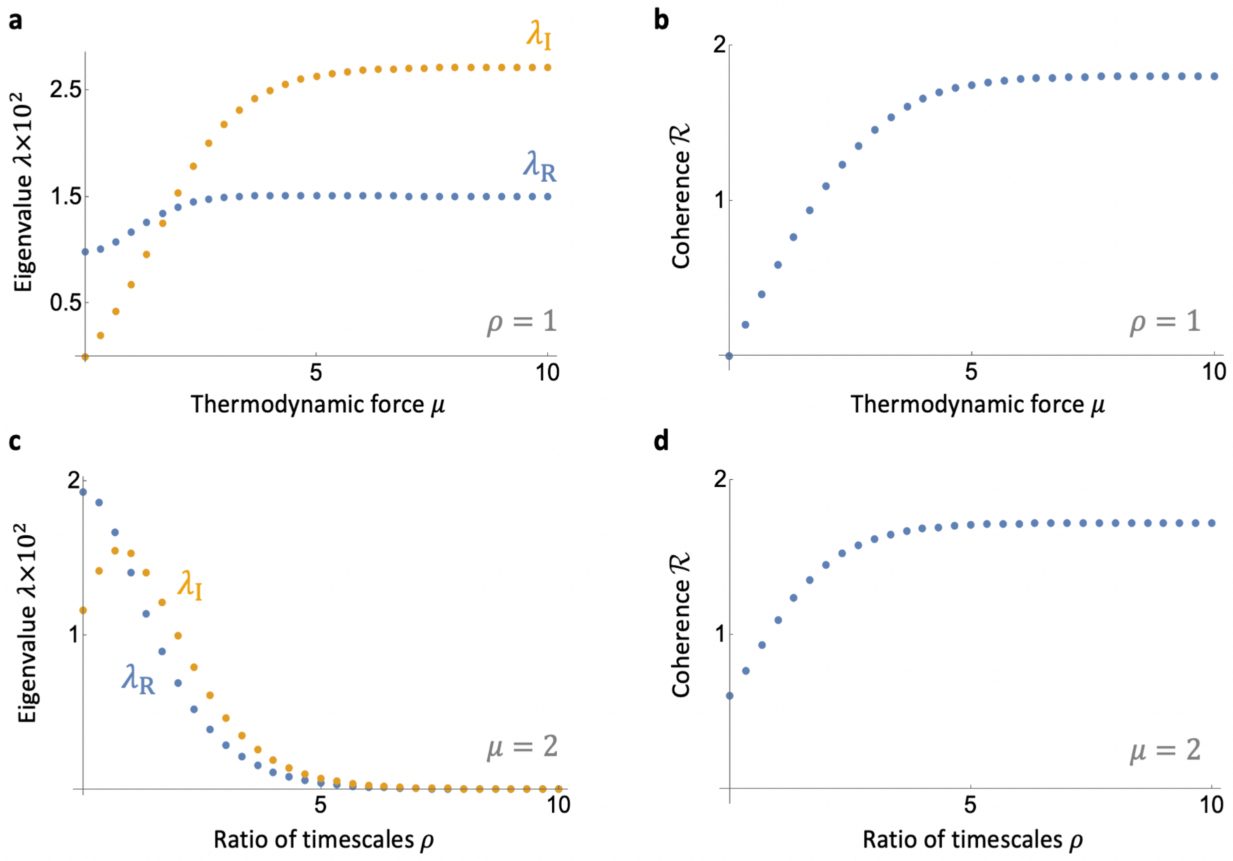

FIG. 8. **Effects of μ and ρ on the first non-zero eigenvalue λ_1 and coherence.** **a**, The absolute value of the real and imaginary parts for λ_1 as a function of μ. λ_{I} increases with μ and approaches a maximum while λ_{R} does not change significantly. **b**, Coherence as a function of μ. $\mathcal{R}$ is almost proportional to λ_{I} given a nearly constant λ_{R}. **c**, The absolute value of the real and imaginary parts of λ_1 as a function of ρ. λ_{R} decreases monotonically while λ_{I} is non-monotonic in ρ. In the regime where λ_{I} decreases, it decreases slower than λ_{R}. **d**, Coherence as a function of ρ, showing a monotonic increase just as in the μ direction. Eigenvalues are given in units of γ_{tot}.

VII. GENERALIZATIONS TO MANY MOLECULES

In this section, we explore generalizations of our single-molecule model to many molecules. In particular, we investigate the higher-dimensional state space formed by two or more KaiC molecules. We further assume that KaiA is a scarce resource and study the resulting state space geometry modified by the constraints introduced by the competition over KaiA. This competition is intrinsically a population-level effect, as all KaiC molecules compete for the same scarce resource to complete the phosphorylation cycle. Finally, we look at the effects of limited or excess amounts of KaiA on the phosphorylation cycles for our single-molecule model or the many-molecule generalization.

We first look at the state space of our model when there are two KaiC molecules in consideration. In this case, the indices $(x_1, x_2, y_1, y_2)_{s_1,s_2}$ completely specify the system of two molecules, where the subscripts on the variables x, y, s denote molecule number. For each phosphorylation level (x_1, x_2, y_1, y_2), there are $4 \times 4 = 16$ internal states that form a tesseract, the 4D generalization of a cube. These internal states repeat themselves in the four directions spanned by the external variables x_1, x_2, y_1, y_2 to form a state space in the shape of a 4D hypercube.

To study population-level effects, we assume that KaiA molecules, which promotes KaiC phosphorylation [12], are a scarce resource that can be depleted. This agrees with experimental observations that when KaiA concentrations drop below a certain point, oscillations are prohibited and KaiC phosphorylation levels remain low [5]. We further assume that each KaiA molecule promotes the T phosphorylation of one KaiC monomer. Since there are six T phosphorylation sites for each KaiC hexamer, we consider the regime $N_{\mathrm{A}} < 12$, where the number of KaiA molecules N_{A} is not enough to fully phosphorylate both molecules. This competition introduces the constraint $x_1 + x_2 \le N_{\mathrm{A}}$. In the following, we consider the geometry of this state space in two cases. In either case, the hypercubic state space of the system is reduced to a hyperprism from the constraint introduced by competition over KaiA. Depending on

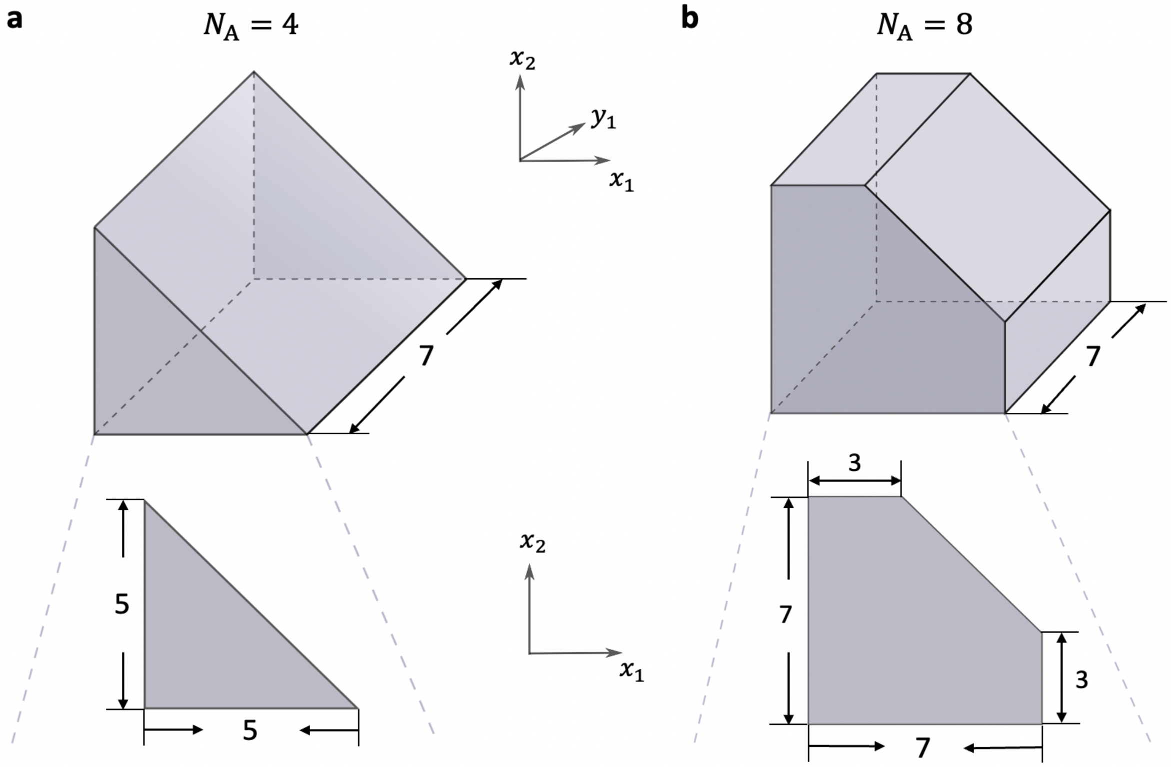

FIG. 9. **Cross sections of the 4D state space for two KaiC molecules when competition for KaiA is taken into account.** **a**, For $N_A = 4$, the constraint $x_1 + x_2 \leq N_A$ from competition over KaiA reduces the state space in x_1-x_2 from a 7×7 square to an isosceles right triangle with side length 5. All side lengths refer to the number of phosphorylation levels contained in that direction. The 3D cross section in y_2 for the complete 4D state space is a right triangular prism, obtained by repeating the isosceles right triangle in y_1. Such geometries come up in the regime $N_A \leq 6$. **b**, For $N_A = 8$, the constraint reduces the state space to an irregular pentagon in x_1-x_2, which results from cutting off a corner from a 7×7 square. The 3D cross section in y_2 is a pentagonal prism that derives from repeating the pentagon in y_1. Such geometries come up in the regime $6 < N_A < 12$.

the amount of available KaiA, the cross-sections of the hyperprism can be different, as illustrated in Fig. 9. Such changes to the geometry of the state space modifies its edges and introduces dependence on phosphorylation levels of different molecules along these new edges, e.g., $x_1 + x_2 = 4$ on the hypotenuse of the triangle in Fig. 9a.

One regime is when $N_A \leq 6$. This is when KaiA can fully phosphorylate one KaiC hexamer at most. In this case, the x_1-x_2 subspace will be reduced from a 2D square to an isosceles right triangle, as illustrated in the lower part of Fig. 9a, taking $N_A = 4$ as an example. To study the shape of the four-dimensional state space, we can look at its 3D cross-sections by holding one coordinate fixed. As shown in Fig. 9a, with a fixed y_2, the state space in x_1-x_2-y_1 space is a right triangular prism, obtained by repeating the right triangle in x_1-x_2 along the y_1 direction. This 3D cross-section is the same for any y_2, which means that this prism repeats itself in the fourth dimension y_2, forming a 4D hyperprism.

In another regime, $6 < N_A < 12$. This is when KaiA can fully phosphorylate one molecule but not enough to phosphorylate both. In Fig. 9b, we illustrate the geometry for the case $N_A = 8$. The x_1-x_2 subspace is an irregular pentagon, formed by removing the upper right corner (an isosceles right triangle) from the 2D square. The 3D cross-section for a fixed y_2 is a pentagonal prism that repeats in y_2 to form another 4D hyperprism, which differs from the above by its different 3D cross-section.

In general, if there are N KaiC molecules, the constraint on the T phosphorylation levels becomes $x_1 + x_2 + ... + x_N \leq N_A$. The state space will be a $2N$-dimensional hyperprism, where high-dimensional "corners" are removed from a $2N$-dimensional hypercube due to the constraint.

When the amount of KaiA is varied, this simple model behaves the same as what is observed in experiments, i.e., limited KaiA leads to sustained low levels of phosphorylation while excess KaiA leads to sustained high levels of phosphorylation [5, 2]. When there is little to no KaiA, states with high T phosphorylation levels are blocked. When

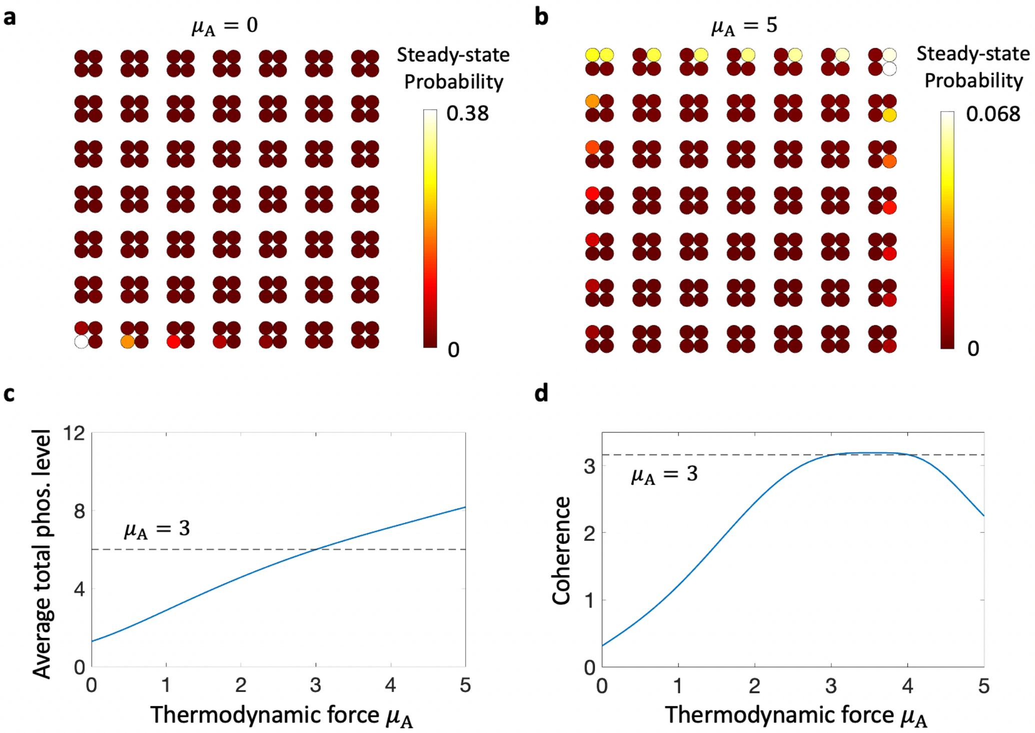

FIG. 10. **The effects of limited or excess amounts of KaiA on the KaiC phosphorylation cycle.** **a**, Steady-state probability distribution in the absence of KaiA. The thermodynamic force for the S $\rightarrow$ E internal transition, μ_A, which characterizes the external driving from interaction with KaiA, is set to be 0. The probability is localized on the lower left corner, corresponding to a hypophosphorylated state for KaiC in the absence of KaiA, consistent with experimental results [5]. In all panels, we take $\mu = 3, \rho = 5$. **b**, Steady-state probability distribution for $\mu_A = 5$. The probability is localized in highly phosphorylated states of KaiC, also consistent with experiments [2]. **c**, Total phosphorylation level of T and S as a function of μ_A, averaged over the steady-state probability distribution. KaiC autodephosphorylates as μ_A decreases with the removal of KaiA, and phosphorylates as μ_A increases with the addition of KaiA. $\mu_A = \mu = 3$ corresponds to the unmodified model as introduced in the main text, whose phosphorylation level is illustrated with a horizontal dashed line. **d**, Coherence as a function of μ_A. Coherent oscillations are inhibited with the removal of KaiA or an excess of KaiA. Coherence for $\mu_A = 3$ is illustrated with a horizontal dashed line.

there is too much KaiA, there is no competition and no constraint whatsoever on the state space, which means that each KaiC molecule oscillates independently without synchronizing. Moreover, the strong driving from KaiA would promote phosphorylation of each molecule (see Fig. 10b), leading to a high overall phosphorylation level. We note that the above discussions have relied on simplifying assumptions on the nature of KaiA-KaiC interactions. A more thorough investigation of a many-molecule model would take into account more realistic properties of KaiA.

Such effects of KaiA can also be captured just by our single-molecule model in the main text. Because the thermodynamic force for the S $\rightarrow$ E internal transition, which we denote as μ_A, characterizes the driving from interaction with KaiA, changes in μ_A correspond to changes in concentrations of KaiA [4]. We keep the slower E $\rightarrow$ S transition rate fixed at $\gamma'_{\rm in}$ and let the S $\rightarrow$ E transition rate depend on μ_A by $\gamma'_{\rm in} e^{\mu_A/k_B T}$. Fig. 10a shows the steady-state probability distribution for $\mu_A = 0$ while μ and ρ are kept the same, which corresponds to complete removal of KaiA. As we can see, the probability is mostly localized in states near the lower left corner, corresponding to low levels of phosphorylation. In contrast, in Fig. 10b we set $\mu_A = 5$, corresponding to excess amounts of KaiA. The probability, in turn, becomes localized in highly phosphorylated states. The average total phosphorylation level (T and S combined) as a function of μ_A is shown in Fig. 10c, which shows increasing levels of phosphorylation as μ_A increases and KaiA is added. For either limited ($\mu_A < 3$) or excess ($\mu_A > 3$) amounts of KaiA, oscillation tends to be attenuated as illustrated by the decrease in coherence in Fig. 10d.

SUPPLEMENTARY REFERENCES

[1] Andre C Barato and Udo Seifert. "Coherence of biochemical oscillations is bounded by driving force and network topology". In: *Physical Review E* 95.6 (2017), p. 062409.

[2] Archana G Chavan et al. "Reconstitution of an intact clock reveals mechanisms of circadian timekeeping". In: *Science* 374.6564 (2021), eabd4453.

[3] Gary K Chow et al. "A night-time edge site intermediate in the cyanobacterial circadian clock identified by EPR spectroscopy". In: *Journal of the American Chemical Society* 144.1 (2022), pp. 184–194.

[4] Terrell L Hill. *Free energy transduction and biochemical cycle kinetics.* Springer-Verlag New York Inc., 1989.

[5] Hakuto Kageyama et al. "Cyanobacterial circadian pacemaker: Kai protein complex dynamics in the KaiC phosphorylation cycle in vitro". In: *Molecular cell* 23.2 (2006), pp. 161–171.

[6] Congxin Li et al. "Circadian KaiC phosphorylation: a multi-layer network". In: *PLoS computational biology* 5.11 (2009), e1000568.

[7] Feng Liu and Katsunori Wakabayashi. "Novel topological phase with a zero berry curvature". In: *Physical review letters* 118.7 (2017), p. 076803.

[8] Jacque Monod, Jeffries Wyman, and Jean-Pierre Changeux. "On the nature of allosteric transitions: a plausible model". In: *Journal of molecular biology* 12.1 (1965), pp. 88–118.

[9] Taeko Nishiwaki and Takao Kondo. "Circadian autodephosphorylation of cyanobacterial clock protein KaiC occurs via formation of ATP as intermediate". In: *Journal of Biological Chemistry* 287.22 (2012), pp. 18030–18035.

[10] Jürgen Schnakenberg. "Network theory of microscopic and macroscopic behavior of master equation systems". In: *Reviews of Modern physics* 48.4 (1976), p. 571.

[11] Evelyn Tang, Jaime Agudo-Canalejo, and Ramin Golestanian. "Topology protects chiral edge currents in stochastic systems". In: *Physical Review X* 11.3 (2021), p. 031015.

[12] Yao Xu, Tetsuya Mori, and Carl Hirschie Johnson. "Cyanobacterial circadian clockwork: roles of KaiA, KaiB and the kaiBC promoter in regulating KaiC". In: *The EMBO Journal* 22.9 (2003), pp. 2117–2126.

[13] Jeroen S van Zon et al. "An allosteric model of circadian KaiC phosphorylation". In: *Proceedings of the National Academy of Sciences* 104.18 (2007), pp. 7420–7425.
