# Supplementary material for: A topological mechanism for robust and efficient global oscillations in biological networks
Source: Nat Commun. 2024 Jul 31;15:6453. doi: 10.1038/s41467-024-50510-x (PMC11291491; doi:10.1038/s41467-024-50510-x)
Supplement: Supplementary file 3 — Description of Additional supplementary file [file 41467_2024_50510_MOESM3_ESM.pdf]

Description of Additional supplementary file

Supplementary Movie 1: A KaiC molecule goes through phosphorylation cycles (right) as the system moves along the edge of the state space in the topological regime (left). The system dynamics is simulated with the Gillespie algorithm for the parameters mu=5, rho=5.
